# Dr. Frank G. Osborne

**Published:** 1911-06

**Authors:** 


Dr. Frank G. Osborne (U. of B. 1868) died suddenly at his home in East Aurora, N. Y., March 2, 1911, aged 66 years.

